# Morphological characterization of indigenous goats in selected districts of West Shewa Zone, Oromia regional State, Ethiopia

**DOI:** 10.1371/journal.pone.0327309

**Published:** 2025-07-09

**Authors:** Kassahun Bekana Kitila, Abera Teshome Aleli, Solomon Shiferaw Tufa

**Affiliations:** Ambo University, Guder Mamo Mezemir Campus, Ambo, Ethiopia; Eskisehir Osmangazi University: Eskisehir Osmangazi Universitesi, TÜRKIYE

## Abstract

This study was conducted in the West Shewa Zone to identify the morphological characteristics of the indigenous goat population. Data were collected from 519 goats, including body measurements and observations. Qualitative data were analyzed descriptively, while quantitative data were analyzed using the GLM procedure. The average flock size per household was 11.30 ± 7.17, with the Ejere district having the largest flocks compared to other districts. Significant differences were observed in most qualitative traits among the goat populations across the four districts. These traits included horn presence, beard presence, coat color pattern, coat hair type, horn shape, head profile, horn orientation, and ear orientation. The majority of goats displayed a patchy coat color, smooth and short hair, straight horn shape, backward horn orientation, straight head profile, and horizontal ear orientation. The overall means for body weight, body length, heart girth, wither height, pelvic height, rump length, rump width, horn length, and ear length were 29.03 ± 3.91 kg, 65.09 ± 3.60 cm, 70.87 ± 3.84 cm, 65.97 ± 3.75 cm, 68.23 ± 3.35 cm, 19.27 ± 2.11 cm, 13.75 ± 1.65 cm, 10.89 ± 2.77, and 14.00 ± 1.37 cm, respectively. Significant differences (p < 0.05) in most body measurements were observed based on sex, and age. In the interaction, some of them were found to be significant differences, while only a few measurements showed differences between districts. Body weight and heart girth exhibited a stronger correlation than other linear body measurements. For female goats, heart girth, body length, and pelvic height significantly influenced body weight predictions, whereas for male goats, only heart girth was a relevant measurement. In conclusion, the observed variations in most morphological traits indicate the necessity of developing selective breeding strategies to enhance economically significant traits in goat farming.

## Introduction

Ethiopia has approximately 52.5 million goats, with almost all of them being indigenous, representing 99.99% of the total goat population [1]. Molecular characterization has identified fourteen goat populations, categorized into seven types. These goats are raised in different production systems and agro-ecological zones [2]. Resource-poor smallholder farmers and pastoralists manage nearly all of these goat populations using traditional and extensive production systems [3].

Goats (*Capra hircus*) are vital to the livelihoods of resource-poor farmers in Ethiopia [4]. Indigenous goats play a significant role in the socioeconomic landscape by providing meat, milk, manure, fibers, and skins. They contribute to income generation, serve as a status symbol, and fulfil various cultural and religious functions [5]. Goat milk is more commonly consumed than sheep milk and is believed to possess superior nutritional and medicinal properties [6]. Smallholders often purchase small ruminants as a means of saving cash for future needs, especially in rural areas where no banking facilities [4].

Indigenous goat populations exhibit valuable traits, such as better performance under low-input conditions and climatic stress, tolerance to infectious diseases and parasites, resilience to heat stress, the ability to survive on woody browse, and the capacity to thrive with infrequent watering. Additionally, goats’ rapid reproduction rates allow them to recover quickly from losses [7–9].

Goat production accounts for 16.8% of the total meat supply [10] and 16.7% of the milk consumed in the country [11]. The average carcass weight of Ethiopian goats is 10 kg, making it the second lowest in sub-Saharan Africa [12]. Despite the widespread distribution and significance of the Ethiopian goat population, its productivity and contribution to both the livelihoods of owners and the national economy remain low. Many scholars attribute the low productivity of the goat population to several factors, including the lack of involvement from farmers and pastoralists in the design and implementation of breeding programs [13–15], poor nutrition, disease prevalence, inadequate breeding strategies, and a limited understanding of the overall production system [16]. Consequently, it is urgent to enhance the productivity of livestock species, particularly goats, to meet the growing demand for animal protein, improve the livelihoods of livestock keepers, and foster the economic development of the country.

Morphological characterization includes both qualitative and quantitative characteristics. Quantitative traits involve the sizes and measurements of an animal’s body and they show a stronger correlation with production traits compared to qualitative traits. For example, body weight and chest girth are direct indicators of body size and related production characteristics. Qualitative traits refer to the observable physical features, such as the shape, color, and overall appearance of animals. Although some of these traits, like coat color, horn shape, and ear length, may not have significant implications for production and service functions, they can be linked to adaptive features. For instance, skin and coat color, ear size, and horn patterns are associated with heat dissipation. Moreover, some qualitative traits serve as a means of animal identification when other methods are not feasible. In these scenarios, qualitative traits hold equal importance to quantitative traits, making them vital elements of phenotypic characterization studies [17].

Furthermore, designing and implementing a breeding program requires a comprehensive understanding of the morphological characteristics of the goat population within a specific environment [18]. Therefore, morphological characterization is crucial for selective breeding and enhancing productivity, as it provides a systematic approach for identifying and selecting desirable traits in animals. Although various research and development efforts have been undertaken in Ethiopia [3], the West Shewa zone has received comparatively less attention, particularly regarding goat genetic resources. The Oromia region is home to 8,425,727 goats, with West Shewa ranking eighth in population size, totalling 264,931 heads [1]. Despite the significant population of indigenous goats in the West Shewa Zone, their morphological characteristics have not been thoroughly documented, and no improvement strategies have been implemented. Thus, this research aims to characterize the morphological traits of the goat population in the study areas and to predict goats’ body weight using linear body measurements, establishing baseline information for future reference.

## Materials and methods

### Description of the study areas

**Fig 1:**
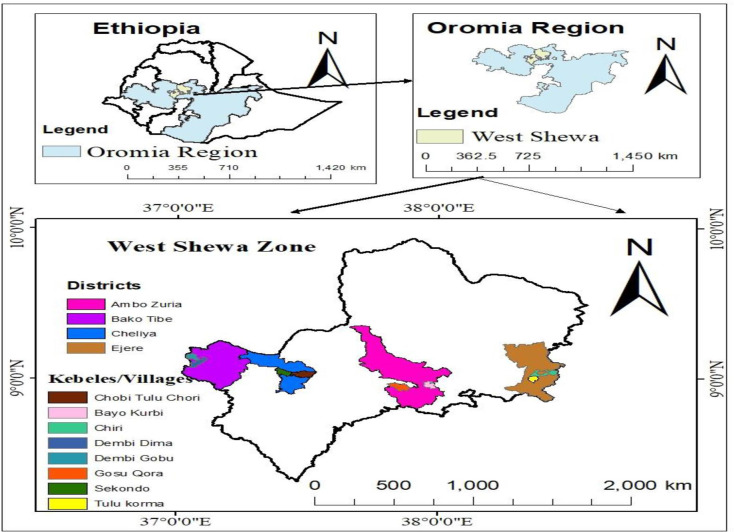
Map of the study area.

West Shewa zone is one of the zones of the Oromia region. Ambo town is the capital town of the zone, which is located 114 km from Addis Ababa, the capital city of the country, Ethiopia, in the west direction, situated between 9° 10′ 0″ N and 37° 50′ 0″. West Shewa has 22 districts. The study was conducted in four districts, namely Ambo, Cheliya, Bako Tibe, and Ejere. The descriptions of districts were depicted in S1 Table. The map of the study area is presented in Fig 1.

### Sampling techniques and sample size

Multi-stage sampling techniques were utilized. Four districts (highland (Cheliya), midland (Ejere, Ambo), and low land (Bako Tibe)) were purposefully selected based on their geographical locations, which influence goat genetic diversity. Within each district, two adjacent villages were chosen, as listed in S1 Table. Goats were selected based on age, as determined by their dentition, and sex. This included goats with young (milk teeth), 1PPI (one pair of permanent incisor), 2PPI (two pair permanent incisor), 3PPI (three pair of permanent incisor), and 4PPI (four pair of permanent incisor) dentition from both sexes. The FAO [18] recommends using 100–300 female animals and 10–30 male animals for breed phenotypic characterization. In line with this recommendation, a total of 466 female goats and 53 male goats were used for morphological characterization.

### Data collection and source

Primary data was collected from farmers and adult goats. Linear body measurements, including body length (BL), heart girth (HG), rump width (RW), rump length (RL), ear length (EL), Scrotal Length (SL) and scrotal circumference (SC) (for males only), were obtained using a flexible tape measure (3 m in length) with a precision of 0.5 cm. The animals were restrained and held in a stable position during this process. Wither height (WH) and pelvic height (PH) were measured using a sliding ruler. The body weight of adult goats was recorded using a portable suspended weighing scale with a capacity of 50 kg and a precision of 200 g. Qualitative data such as sex, coat color, horn characteristics (orientation, shape, presence or absence), ear orientation, beard, and wattle were also observed and documented during the collection of linear body measurements.

### Data analysis

Different statistical analyses were employed based on the nature of the data type. All collected data was filtered and recorded in a Microsoft Excel sheet. Qualitative data gathered through observations of adult goats were analyzed using the Statistical Package for Social Sciences (SPSS, version 20). The chi-square (χ²) test was applied to assess the statistical significance of categorical variables.

General Linear Model procedure from SAS (SAS, version 9.0) was used for the analysis of quantitative traits. Sex, age, district, and the interaction of sex and age were included as fixed variables, while all quantitative traits, except scrotal circumference and scrotum length for females were treated as response variables. Least square means (LSM) along with their corresponding standard errors were calculated for each trait, considering sex, age, district, and the age-by-sex interaction.

The model used for the analyses of least square means in female and male goats, excluding scrotal circumference and scrotum length, is as follows:

**Y**_**ijk**_ **= μ + A**_**i**_ **+ S**_**j**_ **+ D**_**k**_ **+ (AS)**_**ij**_ **+ e**_**ijk**_

Where:

Yijk = the observation of body weight and LBMs excluding scrotum circumference in the i^th^ age group j^th^ sex and k^th^ district

μ = overall mean

A_i_ = the effect of i^th^ age class (i = 1PPI, 2PPI, 3PPI and 4PPI)

S_j_ = the effect of j^th^ sex (j = male, female)

D_k_ = the effect of k^th^ district [k = highland (Cheliya), midland (Ejere, Ambo), low land (Bako Tibe)]

(AS)_ij_ = the interaction effect of i^th^ age class and j^th^ sex

e_ijk_ = random residual error

Model used for the least square mean analysis in males for scrotal circumference was:

**Y_ij_ = μ + Di +Aj + e**_**ij**_

Where:

Y_ij_ = Scrotal Circumference and Scrotal Length

μ = overall mean

D_i_ = the fixed effect of i^th^ district [i = highland (Cheliya), midland (Ejere, Ambo), low land (Bako Tibe)]

A_j_ = the fixed effect of j^th^ age class (j = 1PPI, 2PPI, 3PPI, 4PPI)

e_ij_ = random residual error

Pearson correlation was used to estimate the relationship between body weight and linear body measurements. Multiple linear regressions were also employed to predict body weight from other linear body measurements of goats using a stepwise procedure in SAS 9.0. The criteria used for selecting the best-fitting model included a higher adjusted R² coefficient, a smaller coefficient of variation, a smaller Mallows’ Cp, and a lower root mean square error (RMSE).

#### Multiple linear regression models.

Y_j_ = β_0_ + β_1_X_1_ + β_2_X_2_ + β_3_X_3_ + β_4_X_4_ + β_5_X_5_ + β_6_X_6_ + β_7_X_7_ + β_8_X_8_ + e_j_

In this equation:

Y_j_ = body weight

β_0_ = intercept

The coefficients β_1_, β_2_, β_3_, β_4_, β_5_, β_6_, β_7_, and β_8_ correspond to the independent variables X_1_, X_2_, X_3_, X_4_, X_5_, X_6_, X_7_, and X_8_. These independent variables are defined as follows: body length (BL), heart girth (HG), wither height (WH), pelvic height (PH), rump length (RL), rump width (RW), horn length (HL), and ear length (EL), respectively.

e_j_ = residual error.

### Ethical statement

The data on goat morphology was collected with the permission of the farmers. This study does not impact animal welfare, as it involves only measurement and observation of the animals’ bodies. Therefore, we, the authors, declare that this research does not require ethical approval.

## Result and discussion

### Goat flock size and structure

The flock structures of goats in four districts are summarized in Table 1. The overall average flock size per household across the study areas is 11.30 ± 7.17. This finding is relatively consistent with the results of [19], who reported an average goat flock size of 11.52 ± 9.09 in selected districts of the East Gojjam Zone. Moreover, this report aligns with the findings of [20], which indicate that the flock size of small ruminants in low- and middle-income countries ranges from 5 to 30 heads per farmer.

**Table 1:** Goat flock size of adult and young by sex in selected districts.

Districts	Flock size							
	Young (0PPI)			Adult (≥1PPI)				Total
	Male	Female	Total	Male	Female	Castrated	Total	
Ambo (96)	308	296	604	4	457	2	463	1067
Bako Tibe (108)	237	329	566	18	352	2	372	938
Cheliya (71)	246	231	477	4	348	22	374	851
Ejere (86)	349	316	665	14	471	73	558	1223
Total (361)	1140	1172	2312	40	1628	99	1767	4079

Specifically, the mean goat flock sizes in the Ejere, Ambo, Cheliya, and Bako Tibe districts were 14.22, 11.11, 11.99, and 8.69, respectively. These results indicated that the Ejere district has the highest flock size per household, while Bako Tibe has the lowest. This discrepancy may be attributed to differences in the availability of browsing shrubs, the proximity of districts to markets, and the suitability of the agroecological conditions. Goats thrive in areas with ample shrubs, and farmers located near accessible markets with favourable prices are more inclined to raise goats. Consequently, the Ejere district is characterized by its abundance of shrubs and year-round market accessibility.

As expected, the percentage of young goats (56.7%) exceeds that of adult goats (43.3%). The number of male goats declined as age advanced. Consistent with this finding, [21] also noted that availability of male goats aged ≥ 3PPI was difficult in the North Shewa region of the Amhara Regional State. Farmers may sell male goats before they reach maturity age due to their aggressive nature and vulnerability to theft. Additionally, the higher cost of selling bucks at slaughtering age compared to females may influence this decision.

The overall ratio of mature breeding males to mature breeding females is 1:40, which falls within the recommended range 1:20–1:40 for breeding purposes in an extensive management system [22]. However, this ratio is higher than the findings reported by [19], which indicated a ratio of 1:36 in selected districts of the East Gojjam Zone, and by [23], which reported a ratio of 1:9 in the Borana Pastoral system. When compared between selected districts the ratio varies: Ejere (1:33), Ambo (1:114), Cheliya (1:87), and Bako Tibe (1:19). According to [23] recommendation, male to female ratio can be 1:25 under Community-based breeding program (CBBP), while [22] suggested a range of 1:20–1:40 under different climatic conditions. However, in the Ambo and Cheliya districts of the West Shewa Zone, the ratio of mature males to mature females falls significantly outside of this range. Accordingly, in the Ambo and Cheliya districts, a shortage of breeding bucks has been observed, while this is not the case in Ejere and Bako Tibe districts.

### Morphological characterization

#### Characterization of qualitative traits.

Table 2 presents the qualitative characteristics of male and female goats across the four districts. Overall, the goat population exhibited the following characteristics: 90.9% had horns, 25.4% had wattles, 53.9% had beards, 57.7% had patchy coat colors, 76.5% had short and smooth hair types, 80.1% had straight head profiles, 71.7% had backward horn orientation, 49.3% had horizontal ear shape, and 46.7% had straight horn shape. Most qualitative traits showed significant variation between the districts. The chi-square test (χ²) indicated that most goats’ qualitative traits exhibited significant differences (p < 0.01) among four districts, specifically in horn presence, beard presence, coat color pattern, coat hair type, horn shapehorn orientation, and ear orientation, while a wattle and head profile showed a significant difference at (p < 0.05).

**Table 2:** Qualitative characters of goats’ population of four districts.

Qualitative traits		Districts				Total	$x^{2}$	p-value
		Ejere %	Ambo%	Cheliya%	BakoT %			
Horn	Absence	2.8	3.7	12.3	20.5	9.1	31.7	0.0001
	Presence	98.2	96.3	87.7	79.5	90.9		
Wattle	Absence	79.7	78.5	72.6	65.6	74.6	8.48	0.037
	Presence	20.3	21.5	27.4	34.4	25.4		
Beard	Absence	55.2	28.9	23.3	68.0	46.1	59.8	0.0001
	Presence	44.8	71.1	76.7	32.0	53.9		
Coat color	Plain	38.5	51.1	16.4	52.5	42.3	30.3	0.0001
	Patchy	61.5	48.9	83.6	47.5	57.7		
Coat hair	Short and smooth	85.3	62.2	69.9	86.1	76.5	32.8	0.0001
	Short and Coarse	14.0	32.6	28.8	13.1	21.4		
	Long and Coarse	0.7	5.2	1.4	0.8	2.1		
Head profile	Straight	88.8	77.8	76.7	74.6	80.1	13.4	0.037
	Concave	10.5	22.2	23.3	25.4	19.7		
	Convex	0.7	0.0	0.0	0.0	0.2		
Horn shape	Absence	2.8	4.4	12.3	20.5	9.3	85.2	0.0001
	Straight	60.8	37.0	39.7	45.1	46.7		
	Curved	20.3	23.0	21.9	9.0	18.4		
	Spiral	14.7	34.1	13.7	24.6	22.6		
	Corkscrew	1.4	1.5	12.3	0.8	3.0		
Horn orientation	Absence	2.8	4.4	12.3	19.7	9.1	41.0	0.0001
	Lateral	4.9	5.9	0.0	4.1	4.2		
	Upward	15.4	22.2	6.8	11.5	15.0		
	Backward	76.9	67.4	80.8	64.8	71.7		
Ear shape	Pendulous	13.3	9.6	19.2	22.1	15.4	29.7	0.0001
	Semipendulous	27.3	31.9	50.7	39.3	35.3		
	Horizontal	59.4	58.5	30.1	38.5	49.3		

In the current finding, the majority of goats had horns (90.9%) and beard (53.9); and lacked wattle (74.6) which is in consistence with the previous studies of various goat types: indigenous goats in the West Omo and Bench-Sheko Zone of South-western Ethiopia [24]; in the South West Shewa Zone of Oromia Region [25]; in the Horro Guduru Wollega Zone [26]; on the Woyto-Guji goat type [27]; and in the Gumuz, Agew, Begia-Medir, Bati, Abergelle and Central Abergelle of Amhara Region [28].

In this finding, the coat color of the goat was classified into Plain and Patchy. Plain refers to goats that have only one coat color while Patchy describes goats with two or more coat colors or non-uniform color. Overall patchy coat color goats are a predominant which agrees with the report on indigenous goat in the Gumuz, Agew, Begia-Medir, Bati, Abergelle and Central Abergelle of Amhara Region [28]; but inconsistent with the findings of: Shegaw and Elias [24] in the West Omo and Bench-Sheko Zone of South-western Ethiopia; [25] in the South West Shewa Zone of Oromia Region; [26] in the Horro Guduru Wollega Zone and [27] on the Woyto-Guji goat type. However, majority of the Bako Tibe and Ambo districts goats have plain coats color while the Ejere and Cheliya districts goats have patchy coat colors. This finding indicated that there are different coat colors in the districts. The presence of different coat color may help farmers to improve goat productivity through selection. The finding [29] indicated that coat color in local goats can influence environmental adaptation and productivity, as well as serve as a selection criterion. Moreover, [30] also noted that coat color influences the testicular volume, hormone levels, and semen quality in stressful climatic conditions.

The majority (76.5%) of goat population had short and smooth coat hair type while long and coarse is small proportion (2.1%). Similarly, the previous finding on various goats in the South West Shewa Zone of Oromia Region [25]; in the North Shewa region of the Amhara Regional State [21] indicated that higher proportion of goats had short and smooth coat hair type. The type of coat hair is influenced by the area’s weather conditions, which are linked to adaptation. The finding by [29] and [31], the animals with thick hair, short hair, and less dense coat tended to have a higher capacity to eliminate heat through their respiratory rate and showed less intense heat loss by cutaneous evaporation.

#### Characterization of quantitative traits.

Table 3 presents the quantitative measurements of the goat population. The average body weight of adult goats in the study areas was 29.03 ± 3.91 kg. This finding contrasts with the body weights of indigenous goat populations reported by [24] in the West Omo and Bench-Sheko Zone of Southwestern Ethiopia (27.3 ± 0.18 kg), [25] in the South West-shewa Zone, Oromia region (26.8 ± 0.15 kg), and [32] in the West Hararge Zone, Oromia region (23.9 ± 4.66 kg). The observed difference in body weight may be attributed to variations in breed, management practices, and agro-ecological conditions. Additionally, the overall means for body length, heart girth, wither height, pelvic height, rump length, rump width, horn length, ear length, (scrotum circumference and scrotum length for male only) were 65.09 ± 3.60 cm, 70.87 ± 3.84 cm, 65.97 ± 3.75 cm, 68.23 ± 3.35 cm, 19.27 ± 2.11 cm, 13.75 ± 1.65 cm, 10.04 ± 3.89 cm, 14.00 ± 1.37 cm, 22.24 ± 2.7 cm, and 12.35 ± 1.5 cm respectively.

**Table 3:** Least squares means and standard errors of live body weight (kg) and linear body measurements (cm).

Effects and level	N	BW (kg)		BL (cm)		HG (cm)		WH (cm)		PH (cm)		RL (cm)
LSM ± SD		LSM ± SD		LSM ± SD		LSM ± SD		LSM ± SD		LSM ± SD
Overall	519	29.03 ± 3.91		65.09 ± 3.60		70.87 ± 3.84		65.97 ± 3.75		68.23 ± 3.35		19.27 ± 2.11
CV %		13.77		5.52		5.41		5.69		4.91		10.93
R^2^		0.65		0.62		0.61		0.38		0.42		0.31
		LSM ± SE		LSM ± SE		LSM ± SE		LSM ± SE		LSM ± SE		LSM ± SE
Sex		**		*		**		**		**		**
Female	466	28.11 ± 0.69		63.89 ± 0.67		69.15 ± 0.20		64.73 ± 0.66		67.27 ± 018		19.12 ± 0.11
Male	53	30.58 ± 0.21		65.06 ± 1.19		72.74 ± 1.26		70.23 ± 0.64		71.71 ± 1.10		20.78 ± 0.69
Age		**		**		**		**		**		**
0PPI	87	20.69 ± 0.43		57.83 ± 0.39		63.16 ± 0.41		61.53 ± 0.41		63.60 ± 0.37		18.27 ± 0.23
1PPI	81	25.22 ± 0.79		62.77 ± 0.71		68.75 ± 0.76		66.30 ± 0.75		68.82 ± 0.67		19.78 ± 0.42
2PPI	71	29.19 ± 1.43		63.57 ± 1.30		72.28 ± 1.38		66.75 ± 1.35		69.88 ± 1.20		20.24 ± 0.76
3PPI	75	34.25 ± 2.02		67.31 ± 1.83		74.81 ± 1.92		69.80 ± 1.88		71.18 ± 1.68		20.59 ± 1.06
4PPI	205	34.47 ± 2.01		69.30 ± 1.82		75.74 ± 1.94		73.02 ± 1.89		73.95 ± 1.70		20.88 ± 1.07
Districts		Ns		Ns		*		Ns		**		
Ambo	135	28.68 ± 0.74		65.20 ± 0.71		70.27 ± 0.72		67.20 ± 0.70		69.03 ± 0.63		20.87 ± 0.39
Bako	124	28.88 ± 0.70		64.45 ± 0.67		70.95 ± 0.67		68.07 ± 0.66		70.49 ± 0.59		20.76 ± 0.37
Cheliya	98	29.03 ± 0.77		64.18 ± 0.74		71.76 ± 0.74		67.74 ± 0.73		69.99 ± 0.65		20.11 ± 0.41
Ejere	162	28.45 ± 0.72		64.07 ± .69		70.78 ± 0.70		66.91 ± 0.68		68.42 ± 0.61		18.06 ± 0.38
Sex by age		Ns		*		Ns		**		**		Ns
Female, 0PPI	45	19.15 ± 0.59		56.62 ± 0.55		62.39 ± 0.58		59.93 ± 0.57		62.75 ± 0.51		17.91 ± 0.31
Female, 1PPI	74	23.93 ± 0.46		61.70 ± 0.42		66.73 ± 0.45		63.24 ± 0.44		65.89 ± 0.39		18.69 ± 0.25
Female, 2PPI	138	26.94 ± 0.46		63.56 ± 0.44		69.49 ± 0.46		65.65 ± 0.45		68.41 ± 0.41		19.47 ± 0.25
Female, 3PPI	74	30.22 ± 0.45		65.14 ± 0.4		71.5 ± 0.45		66.27 ± 0.44		68.58 ± 0.39		19.59 ± 0.24
Female, 4PPI	204	34.66 ± 0.28		69.77 ± 0.77		75.61 ± 0.28		68.55 ± 0.27		70.70 ± 0.24		19.96 ± 0.15
Male, 0PPI	42	22.26 ± 0.61		59.04 ± 0.56		63.92 ± 0.59		63.13 ± 0.58		64.45 ± 0.52		18.62 ± 0.33
Male, 1PPI	7	26.46 ± 1.46		63.83 ± 1.37		70.76 ± 1.45		67.85 ± 2.66		71.36 ± 2.38		20.86 ± 0.80
Male, 2PPI	2	31.46 ± 2.78		63.59 ± 2.56		74.00 ± 3.85		69.35 ± 1.42		71.66 ± 3.36		21.01 ± 1.41
Male, 3PPI	2	36.36 ± 3.93		71.64 ± 1.81		77.52 ± 2.72		75.41 ± 1.42		75.53 ± 1.27		21.69 ± 1.06
**Effects and level**	**N**		**RW (cm)**		**HL (cm)**		**EL (cm)**		**SC (cm) (N = 53)**		**SL (cm) (N = 53)**	
			LSM ± SD		LSM ± SD		LSM ± SD		LSM ± SD		LSM ± SD	
Overall	519		13.75 ± 1.65		10.89 ± 2.77		14.00 ± 1.37		22.24 ± 2.7		12.35 ± 1.5	
CV %			12.00		25.38		9.84		12.29		11.92	
R^2^			0.37		0.41		0.14		0.13		0.18	
			LSM ± SE		LSM ± SE		LSM ± SE		LSM ± SE		LSM ± SE	
Sex			Ns		**		Ns		NA		NA	
Female	466		13.47 ± 0.09		10.06 ± 0.16		13.94 ± 0.07					
Male	53		13.62 ± 0.54		12.26 ± 0.49		13.79 ± 0.45					
Age			**		**		Ns		Ns		Ns	
0PPI	87		11.49 ± 0.18		7.78 ± 0.32		13.40 ± 0.15		22.3 ± 0.4		12.2 ± 0.2	
1PPI	81		13.97 ± 0.32		9.76 ± 0.40		13.61 ± 0.27		22.5 ± 1.2		12.6 ± 0.6	
2PPI	71		13.57 ± 0.59		11.22 ± 0.42		13.25 ± 0.50		22.5 ± 2.0		13.3 ± 0.5	
3PPI	75		13.87 ± 0.83		12.52 ± 0.42		13.69 ± 0.70		28.0 ± 2.8		14.7 ± 0.1	
4PPI	205		14.82 ± 0.84		14.52 ± 0.34		15.35 ± 0.69					
Districts			**		Ns		**		Ns		Ns	
Ambo	135		13.81 ± 0.31		11.09 ± 0.32		13.37 ± 0.26		23.7 ± 1.2		13.2 ± 0.7	
Bako	124		13.84 ± 0.29		11.36 ± 0.34		14.49 ± 0.24		23.8 ± 1.1		13.8 ± 0.6	
Cheliya	98		13.64 ± 0.31		11.32 ± 0.36		13.81 ± 0.27		24.9 ± 1.3		13.3 ± 0.7	
Ejere	162		12.89 ± 0.30		11.56 ± 0.30		13.77 ± 0.25		22.7 ± 1.0		12.6 ± 0.6	
Sex by age			Ns				Ns		NA		NA	
Female, 0PPI	45		11.61 ± 0.25				13.38 ± 0.21					
Female, 1PPI	74		13.14 ± 0.19				13.73 ± 0.52					
Female, 2PPI	138		13.65 ± 0.20				13.96 ± 0.16					
Female, 3PPI	74		14.12 ± 0.19				14.21 ± 0.17					
Female, 4PPI	204		14.84 ± 0.12				14.40 ± 0.10					
Male,0PPI	42		11.38 ± 0.26				12.29 ± 0.98					
Male,1PPI	7		13.49 ± 1.17				13.42 ± 0.21					
Male, 2PPI	2		14.80 ± 0.63				13.48 ± 0.53					
Male, 3PPI	2		15.51 ± 1.67				14.86 ± 0.69					

BW = Body weight, BL = Body length, HG = Heart girth, WH = Wither Height, PH = Pelvic Height, RL = Rump Length, RW = Rump Width, HL = Horn length, EL = Ear Length, SC = Scrotum circumference, SL = Scrotum Length, CV = Coefficient of variance, R^2 ^= R-square, 1PPI = one pair of permanent incisor, 2PPI = Two pair of permanent incisor, 3PPI = Three pair of permanent incisor, LSM = least square mean, SD = Standard deviation, SE = Standard Error, Ns = Not significant at p = 0.05, NA = Not available.

**Districts effect**: At least (p < 0.05), there are significant differences among districts in heart girth, pelvic height, rump width, and ear length. This difference may be related to agroecological effects and management. The environmental factors impacting livestock productivity include the size and productivity of the grazing land [27].

**Sex effect:** Body weight and all linear body measurements, except for rump width and ear length, were influenced by sex. Male body weight is generally expected to be higher than that of females at the same age due to hormonal effects. Specifically, the release of androgens—which are known to stimulate growth and weight—in male animals occurs after the testes have fully developed (Frandson and Elmer, 1981) cited by [33]. In the study area, adult bucks available were only ≤ 2PPI, while the does’ ages ranged from 1PPI to 4PPI. This observation aligns with the finding noted the difficulty in finding males aged 3PPI or older in North Shewa of the Amhara regional state [21]. For example, when comparing a doe aged 4PPI to a buck aged 1PPI, the doe exhibits greater body weight than the male. Therefore, it is recommended to compare individuals of the same age across different sexes to better understand the impact of sex on body weight.

**Age effect**: Age as estimated from dentition has a significant effect (p < 0.05) on body weight and all linear body measurements except ear length of the goat population in the study areas. It is expected that young goats have lower body weight and shorter linear body measurements than adult goats. As the goats aged, their body weight and linear measurements increased. This aligns with the findings of regarding the goat population in the North Shewa Zone [21], on indigenous goats in the Nuer Zone [33], and focused on Woyto-Guji goats [27]. The dentition of 3PPI and 4PPI goats indicates they have reached maturity and finished growth. In contrast, the milk teeth of 1PPI and 2PPI goats are still in the growing stage. Female goats at the 1PPI and 2PPI stages have lower body weight and shorter linear body measurements compared to those at the 3PPI and 4PPI stages. This disparity may be attributed to the fact that the feed consumed by 1PPI and 2PPI goats is allocated for maintenance, growth, production, and reproduction, while the feed for 3PPI and 4PPI goats is not utilized for growth.

**Sex by age interaction**: It has a significant effect (p < 0.05) on body length, wither height and pelvic height. The proportion of females and males under the same age difference may affect the validity of the test.

### Correlation of body weight and linear body measurements

Phenotypic correlation is influenced by both genetic and environmental factors. In the study areas, measurements of body weight and heart girth exhibited a stronger correlation than other linear body measurements (r = 0.902). Furthermore, the correlation between body weight and body length is 0.853, or 85.3%, indicating a significant (p < 0.0001) relationship between these two measurements. This finding aligns with correlation coefficients in the studies range from r = 0.81 to 0.96 [26,34,35]. Therefore, it is possible to estimate a goat’s body weight by measuring its heart girth and body length. This suggests that factors influencing body weight are likely to also affect heart girth, body length and vice versa. These influencing factors may be either genetic or environmental. Pelvic and wither height also exhibit a strong positive correlation, with a correlation rate of 84.5%. Highly correlated traits are crucial for reducing selection costs in multiple trait selection, as the selection of one trait can influence the selection of other characteristics. Generally, the relationships between body weight and various linear body measurements are classified as positively correlated traits as indicated in Table 4.

**Table 4:** Pearson Correlation between Goats’ Body Measurements.

	BW	BL	HG	WH	PH	RL	RW	HL	EL
BW	1.000	0.0001	0.0001	0.0001	0.0001	0.0001	0.0001	0.0001	0.0001
BL	0.853	1.000	0.0001	0.0001	0.0001	0.0001	0.0001	0.0001	0.0001
HG	0.902	0.806	1.000	0.0001	0.0001	0.0001	0.0001	0.0001	0.0001
WH	0.709	0.706	0.723	1.000	0.0001	0.0001	0.0001	0.0001	0.0001
PH	0.714	0.691	0.724	0.845	1.000	0.0001	0.0001	0.0001	0.0001
RL	0.379	0.418	0.344	0.413	0.465	1.000	0.0001	0.0001	0.0002
RW	0.610	0.608	0.655	0.518	0.586	0.422	1.000	0.0001	0.0001
HL	0.505	0.517	0.515	0.420	0.384	0.207	0.333	1.000	0.0001
EL	0.314	0.290	0.323	0.298	0.326	0.164	0.242	0.173	1.000

### Multiple linear regression analysis

Multiple linear regressions between body weight and linear body measurements are essential for predicting animals’ body weight when using a weighing scale is impractical. Predictions can be made from easily measurable linear body measurements, such as heart girth, body length, wither height, and pelvic height. Understanding animals’ body weight is crucial for effective management including feeding, health care, marketing, and reproduction [32].

The stepwise procedure for multiple linear regression models used to predict the body weight of goats based on linear body measurements is detailed in Table 5. The coefficient of determination (R²) indicates the proportion of total variability explained by the model. For female goats, heart girth, body length, and pelvic height measurement significantly affect body weight prediction (p < 0.0001). Heart girth has a significant effect (p < 0.0001) on predicting body weight for male goats. Therefore, heart girth is a best predictor of body weight for both sexes than other linear body measurements. This finding is consistent with the study on Nguni goats [36] and on Hararghe highland goats [32], both of which indicated that heart girth and body length are good predictors of body weight.

**Table 5:** Stepwise procedure of multiple linear regressions for male and female goats.

Model		Parameters			CV	R^2^	RMSE	Mallows’ Cp	p-value
Male	I (β0)	β1	β2	β3					
HG	−24.23	0.73			10.50	0.77	2.50	3.12	0.0001
Female									
HG	−42.91	1.02				0.81	8.30	194.49	0.0001
HG + BL	−48.05	0.68	0.45			0.86	5.97	11.00	0.0001
HG + BL + PH	−51.66	0.64	0.42	0.12	8.17	0.87	5.87	4.02	0.0001

I = Intercept, CV = Coefficient Variation, R^2^ = R-square, RMSE = Root Mean Square Error, HG = Heart Girth, BL = Body Length, PH = Pelvic Height.

Multiple Regression models for predicting body weight of female and male goat population from linear body measurements:

Y = response variable (body weight)

Heart girth (HG), body length (BL), wither height (WH), pelvic height (PH), rump length (RL), rump width (RW), horn length (HL), ear length (EL) = explanatory variable:

Stepwise procedure of multiple linear regression models for male goats

Y = -24.23 + 0.73HG--------------------------Step 1

Stepwise procedure of multiple linear regression models for female goats

Y= -42.91+1.02HG---------------------------------Step 1

Y=-48.05+0.68HG+0.45BL------------------------Step 2

Y= -51.66+0.64HG+0.42BL+0.12PH-------------Step 3

No other variable met the 0.05 significance level for entry into the model

The best model for predicting body weight from linear body measurements features a smaller coefficient of variation, a lower Mallows’ Cp, a higher R-squared valued, and a lower root mean square error (RMSE). Accordingly, Table 5 results indicated that Y = −24.23 + 0.73HG for males and Y = −51.66 + 0.64HG + 0.42BL + 0.12PH for females is the best body weight predictor.

## Conclusion and recommendation

### Conclusion

The West Shewa zone is the most prominent agro-ecological area for goat farming. Among the selected districts, Ejere district has the largest goat population. The majority of the goats in the study areas exhibit a straight head profile, straight horns with a backward orientation, horizontal ears, short and smooth hair, and patchy coat colors. Most goats do not have wattles. Sex significantly affected various measurements, including body weight, body length, heart girth, pelvic height, rump length, horn length, and ear length. Additionally, age also influenced body weight, body length, heart girth, wither height, pelvic height, rump length, rump width, and horn length. Notably, body weight and heart girth measurements showed a stronger correlation (r = 0.902) than the other linear body measurements. Heart girth measurement is the best predictor of live body weight for both male and female goats. In conclusion, the observed variations in most morphological traits indicate the necessity of developing selective breeding strategies to enhance economically significant characteristics in goat farming.

### Recommendations

Based on the findings from the morphological characterization of indigenous goats in selected districts of the West Shewa zone, the following recommendations can be made:

- A community-based breeding program should be implemented to address the shortage of breeding bucks and to conserve the local indigenous goat population in the study areas.
- Strengthening farmers’ ability to develop simple and low-cost assessments for feeding management, disease treatment, and marketing decisions for goat flocks relies on the strong relationship between body weight and linear body measurements, particularly when using a weighing scale is impractical.
- Farmers should receive training on breeding strategies for indigenous goats to improve productivity.

## Supporting information

S1 Table Description of the study districts. (DOCX)
