# Supplementary material for: Morphological characterization of indigenous goats in selected districts of West Shewa Zone, Oromia regional State, Ethiopia
Source: PLoS One. 2025 Jul 9;20(7):e0327309. doi: 10.1371/journal.pone.0327309 (PMC12240294; doi:10.1371/journal.pone.0327309)
Supplement: S1 Table — (DOCX) [file pone.0327309.s001.docx]

**Table 1S: Description of the study districts**

| Districts | Villages | Altitude | Rainfall | Temperature | Distance from Addis Ababa | Distance from Zone | Land coverage | Goat population | Source |
| --- | --- | --- | --- | --- | --- | --- | --- | --- | --- |
| Bako Tibe | Dembi Dima  Dembi Gobu | 1610-2500m | 800-1200mm | 13.8-27.8^0^c | 250km to west | 136km to west | 64469 ha | 15981 | BTDAO, 2022 |
| Cheliya | Sekondo  Chobi | 1700-3051m | 1150-1400mm | 7-25^0^c | 200km to west | 86km to west | 50965 ha | 19375 | CHDAO, 2022 |
| Ambo | Bayo Kumbi  Gosu Kora | 1380- 3030m | 900– 1200mm | 15-29 ^0^c | 114km to west | - | 86593 ha | 178151 | ADAO, 2022 |
| Ejere | Tulu korma  Tulu Chiri | 2060- 3185m | 900- 1200mm | 22- 28^0^c | 44km to west | 70km to east | 55328 ha | 36000 | EDAO, 2022 |

BTDAO = Bako Tibe District Agricultural Office, CHDAO = Cheliya District Agricultural Office, ADAO = Ambo District Agricultural Office, EDA = Ejere District Agricultural Office
